# Probing fibronectin–antibody interactions using AFM force spectroscopy and lateral force microscopy

**DOI:** 10.3762/bjnano.6.118

**Published:** 2015-05-15

**Authors:** Andrzej J Kulik, Małgorzata Lekka, Kyumin Lee, Grazyna Pyka-Fościak, Wieslaw Nowak

**Affiliations:** 1 Laboratoire de la Physique de la Matière Vivante, Ecole Polytechnique Fédérale de Lausanne (EPFL), CH-1015 Lausanne, Switzerland; 2 Institute of Nuclear Physics, Polish Academy of Sciences, Radzikowskiego 152, 31-342 Kraków, Poland; 3 Department of Histology, Jagiellonian University Medical College, Kopernika 7, 31-034 Kraków, Poland,; 4 Institute of Physics, Faculty of Physics, Astronomy and Informatics, Nicolaus Copernicus University, Grudziądzka 5/7, 87-100 Toruń, Poland

**Keywords:** fibronectin, lateral force microscopy, molecular recognition, torsional forces calibration

## Abstract

The first experiment showing the effects of specific interaction forces using lateral force microscopy (LFM) was demonstrated for lectin–carbohydrate interactions some years ago. Such measurements are possible under the assumption that specific forces strongly dominate over the non-specific ones. However, obtaining quantitative results requires the complex and tedious calibration of a torsional force. Here, a new and relatively simple method for the calibration of the torsional force is presented. The proposed calibration method is validated through the measurement of the interaction forces between human fibronectin and its monoclonal antibody. The results obtained using LFM and AFM-based classical force spectroscopies showed similar unbinding forces recorded at similar loading rates. Our studies verify that the proposed lateral force calibration method can be applied to study single molecule interactions.

## Introduction

The invention of atomic force microscopy (AFM) opened up new areas of research as it can probe various biological structures with nanometer resolution, including images of DNA [1], proteins [2], and cellular surfaces [3–4]. Apart from the imaging aspect, AFM can also be applied to probe molecular interactions with a force resolution of tenths of pN. This method enables the measurement of the strength of the interaction forces between a single pair of molecules [5–7] such as biotin–avidin, biotin–streptavidin [8], or lectin–carbohydrate [9]. Direct measurements of intermolecular forces for complementary DNA strands have been carried out as well [10].

Protein–antibody interactions are of particular interest in immunochemical-based diagnosis [11]. Therefore, studies of the interaction forces provide valuable insight into the mechanisms behind biological interactions. AFM allows for a unique opportunity to probe the properties of individual ligand–receptor complexes and provides details on the structure and behavior of single molecules in conditions close to natural ones [6–9]. This technique provides several advantages over traditional methods including, for example, characterization of states that are undetectable in ensemble approaches where the average value of a property is monitored. Thus, any improvements in the cantilevers or measurement methodologies leading to an increase in speed, resolution, and/or force sensitivity are essential in nanotechnology development.

In the majority of AFMs, the cantilever deflection is recorded by an optical detection system composed of a laser and a position-sensitive photodiode having an active area divided into four quadrants. The deflection (referred to here as the normal deflection) and torsion (referred to here as the lateral deflection) signals are determined as follows: the signal difference between the two upper and lower quadrants is a measure of the normal deflection, while torsion of the cantilever is represented as the signal difference between the two left and two right quadrants.

For an AFM working in force spectroscopy mode (referred to here as AFM-FS), the interactions forces are determined from the analysis of force curves. A force curve represents the dependence between the deflection of the AFM cantilever in the direction perpendicular (normal) to the surface and a relative position on a sample. In the AFM-FS measurement, force curves are recorded point-by-point, requiring a precise but tedious and very time consuming procedure.

Lateral force microscopy (LFM), also called friction force microscopy (FFM) is another operational mode in a standard AFM instrument working in contact mode [12]. In LFM, the cantilever is moved laterally over the investigated surface. In this case, the interaction forces cause cantilever torsion and thus, instead of a perpendicular deflection, the torsion is recorded as a function of the relative position on the sample. To determine the magnitude of the interaction forces, the force curves obtained for torsion can be processed in the same way as force curves obtained in AFM-FS. The two main advantages of the LFM mode are: a much higher unbinding speed applied to bonds and faster measurements. The higher velocity that is used during the LFM experiments to break the bonds enables deeper parts of the energy landscape of the studied molecular complex to be probed. The use of LFM working in the “continuous” line scan mode might help to more quickly probe molecular interactions and should give quantitative estimates of interaction forces.

Wider applications of the fast LFM method are hampered by impediments in the quantitative determination of the force value. In contrast to AFM-FS, this requires a reliable and repetitive calibration procedure. Irrespective of the applied experimental methodology (AFM-FS or LFM), the calibration proceeds through similar steps: (1) determination of the photodiode sensitivity converting the measured signal (in V) into a displacement of the cantilever (in nm) and (2) estimation of the cantilever spring constant used to deliver force (in nN). The calibration of normal deflection, typical for AFM-FS, poses no problem and is based on a well-known procedure utilizing thermal excitations of the cantilever. To date, there are only a few methods that can be used for the lateral force calibration [13–14], but unfortunately, none are fully reliable. For example, recently, Dendzik et al. proposed that the stretching of a reference single molecule (e.g., dextran) could be used to determine the normal and lateral AFM cantilever calibration [15]. Although this new method presents a clear improvement over previous attempts to obtain a reliable calibration for lateral measurements, it requires special hardware. Similarly, the method proposed very recently by Wang and Gee requires an additional calibration tool [16], which may be troublesome as well.

In the presented work, we propose an alternative method for torsion force calibration. It is based on the cantilever deflection measurements carried out during the lateral scanning over a rectangular, reference cantilever with a known normal spring constant. Our method is relatively simple to use, fast, and it does not require any special equipment. In order to verify the extent to which the LFM is suitable for probing molecular interactions, we have measured interaction forces between protein fibronectin (FN) and monoclonal antibody against FN (FN-Mab) using both AFM-FS and LFM techniques. The relation between the unbinding force and the loading rate obtained by AFM-FS was compared with the corresponding relation gathered using LFM. Our results show that the new calibration method has potential for applications in LFM quantitative investigations of intermolecular interactions.

## Results

### Converting torsion into force units

The calibration of the force that acts perpendicular to the investigated surface requires the knowledge of the normal cantilever spring constant and normal photodetector sensitivity. The nominal, normal spring constant was controlled by monitoring the resonant frequency of a thermally excited cantilever [17], carried out before functionalization with an antibody. Since the resonance frequency of the cantilever was almost constant (8.73 ± 0.07 kHz), the nominal value of the spring constant was used to measure the force value. The photodetector sensitivity (referred to here as normal PSD sensitivity) was determined from the slope of the force curve in the region of tip contact with the reference glass surface (for type C cantilevers, the normal PSD sensitivity was 22.1 ± 3.5 nm/V).

Analogous to the normal force measurement, the lateral force (inferred from the torsion of the cantilever) was determined by multiplying the recorded signal (measured in V) by the torsional spring constant and lateral photodetector sensitivity. Both parameters are difficult to estimate using known methods [14,18], so here we propose a simple, alternative method that allows the measured signal (in V) to be directly converted into force units. The conversion factor is referred to here as the lateral PSD sensitivity.

**Figure 1:**
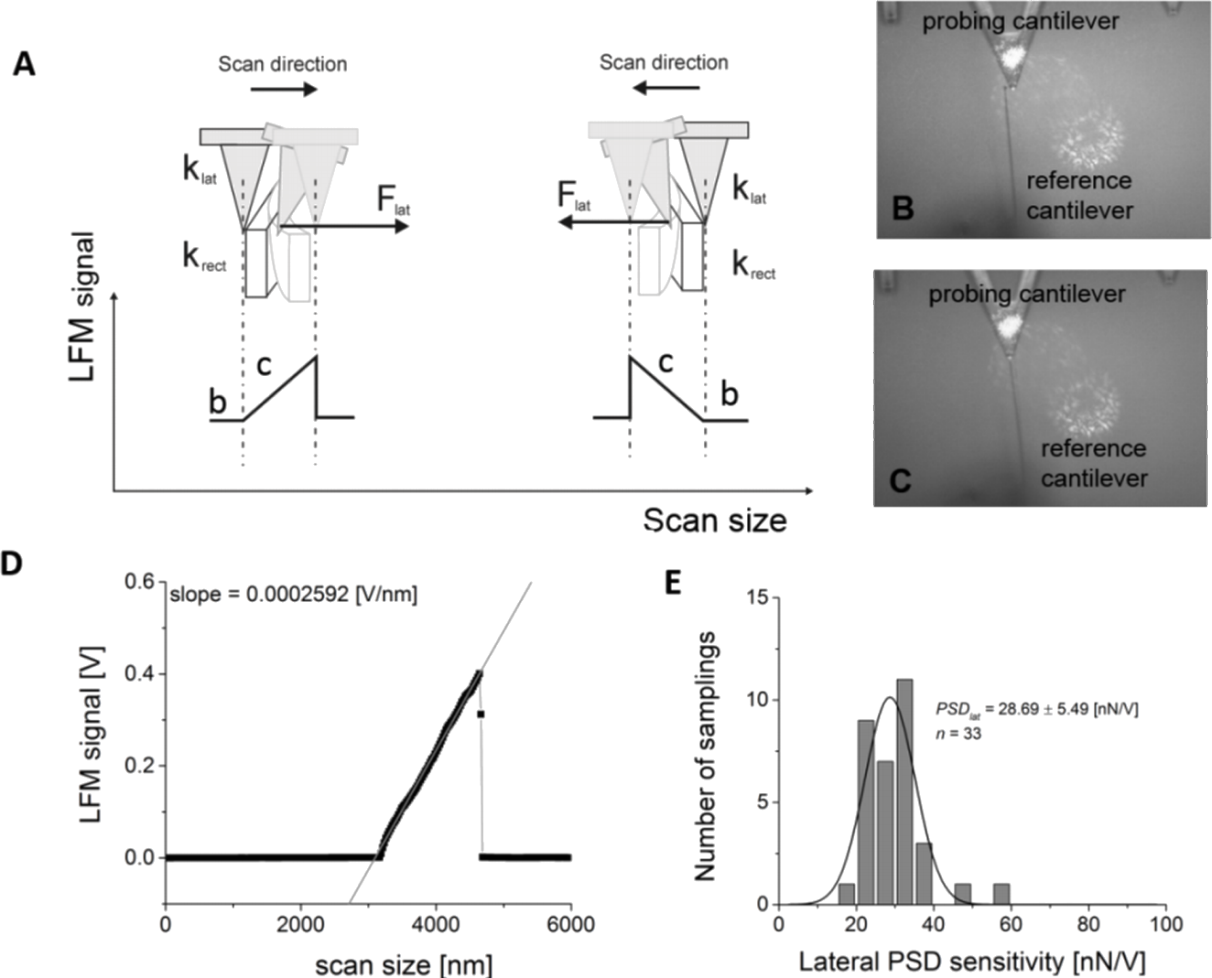
The lateral force calibration concept. (A) An illustration of the calibration approach. (B,C) Images recorded during the scanning of a reference cantilever with a probe MLCT type C cantilever. (D) A calibration curve recorded using the proposed calibration approach. (E) The distribution of the calibration factors (lateral PSD sensitivity) expressed in nN/V.

The calibration concept is presented in Figure 1. Two cantilevers were used: a reference and a probe. The uncharacterized probe cantilever scans over the rectangular reference cantilever of known normal spring constant. The choice of the rectangular shape of the reference cantilever was motivated by the fact that it is easy to bend such a cantilever (one can easily access the end of a cantilever mounted perpendicularly). In our experiments, rectangular cantilevers (micro lever for contact and tapping mode (MLCT), type B) with a nominal spring constant of 0.02 N/m were used.

The lateral signal was recorded while scanning in both directions, as presented schematically in Figure 1A. The optical images of the calibration steps are shown in Figure 1B,C. They were recorded while scanning the reference cantilever with a triangular probe cantilever (MLCT type C, 0.01 N/m). The contour of the LFM signal has a characteristic pyramidal shape that reflects the signal recorded during bending of the reference cantilever (Figure 1D). Each scan consists of 2048 points, recorded over a distance of 6000 nm. From the slope, a lateral calibration factor can be determined by fitting a straight line. A linear regression gives a goodness of fit in the range of 0.992–0.998. The calculated slope was then converted into nN/V by inverting it and multiplying by the known cantilever spring constant of the reference cantilever. From the distribution of the calibration factors (Figure 1E), a mean value of 28.7 ± 5.5 nN/V was calculated, giving a ≈19% accuracy. This is also a measure of the reproducibility (33 cantilevers were calibrated in this manner).

### Surface topography

**Figure 2:**
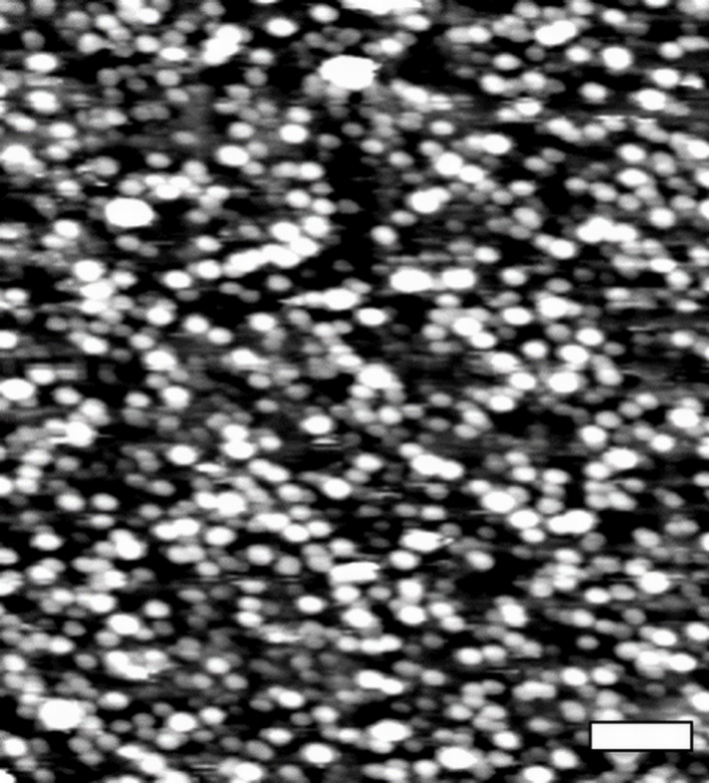
Topography image of FN deposited on a mica surface recorded while scanning in contact mode AFM with a bare AFM tip. Scale bar: 1 µm.

To verify whether the functionalization of a mica surface gave an expected layer of fibronectin molecules, the surface topography was recorded using a bare (non-functionalized) cantilever. As shown in Figure 2, the fibronectin molecules had a regular globular shape and were uniformly distributed over the entire scanned area. The FN height ranged from 0.5 to 3.5 nm with a mean value of 2.4 ± 0.9 nm.

### Dependence of friction force on normal load

The frictional interaction between surfaces observed on the macroscale is typically modelled using Amonton’s law, where a frictional force is linearly dependent on a load force. The proportionality factor is the constant friction coefficient. To verify whether any friction force is observed between the FN-coated surface and the FN-Mab-functionalized AFM probe, the LFM images were recorded as a function of the load force from 0.1 to 4 nN.

**Figure 3:**
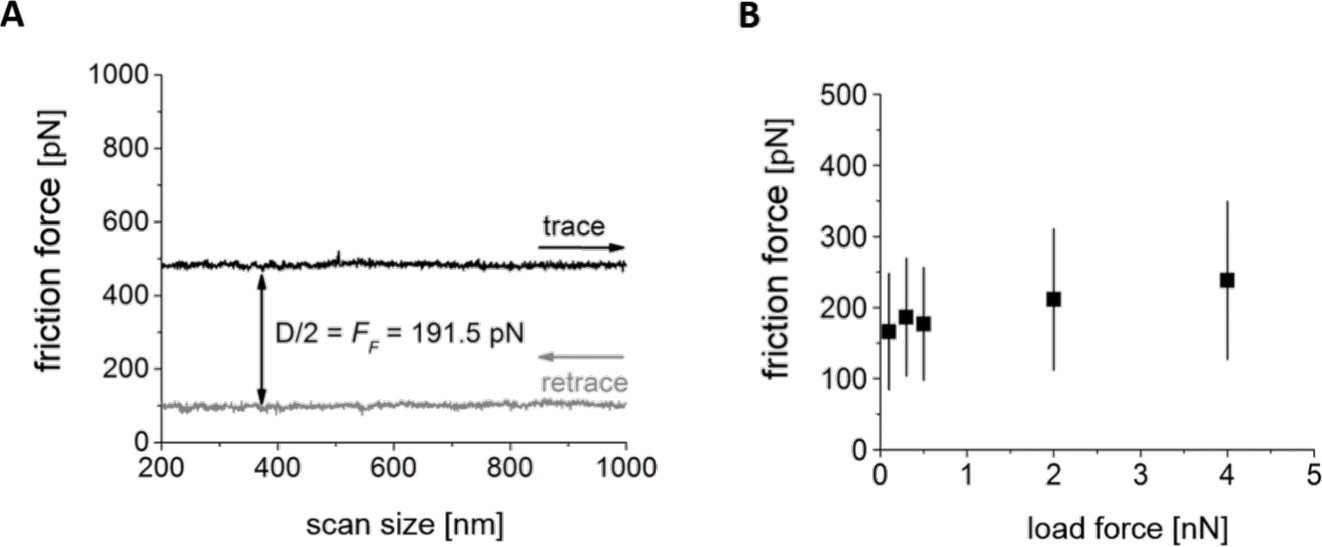
(A) Friction loop determined for a load of 2 nN. The difference, D, between the lateral signal recorded for the trace and retrace modes divided by 2 determines the friction force. (B) The dependence of the friction force on normal load (friction force given as the mean ± standard deviation).

The friction force value was determined by subtracting the mean values calculated separately for scans running in two opposite directions (i.e., trace and retrace) along the same path (see Figure 3A). Thus, the mean value of the friction force was calculated from the distribution of the friction force recorded during such a scan in both directions. The width of the distribution represents the measurement error. In the presented measurements, only a weak dependence of the friction force on the normal load was observed (Figure 3B), therefore, all further measurements of the FN-Mab interaction forces were carried out at the set point of 0.1 nN.

### Unbinding force determination

To study the unbinding process using both LFM and the AFM-based classical force spectroscopies, the measurements of the unbinding force between fibronectin and monoclonal antibody were carried out. We assume that independent of the applied unbinding direction (i.e., normal or lateral), both methods (AFM-FS and LFM) provide similar values of the interaction force at similar loading rates.

**Figure 4:**
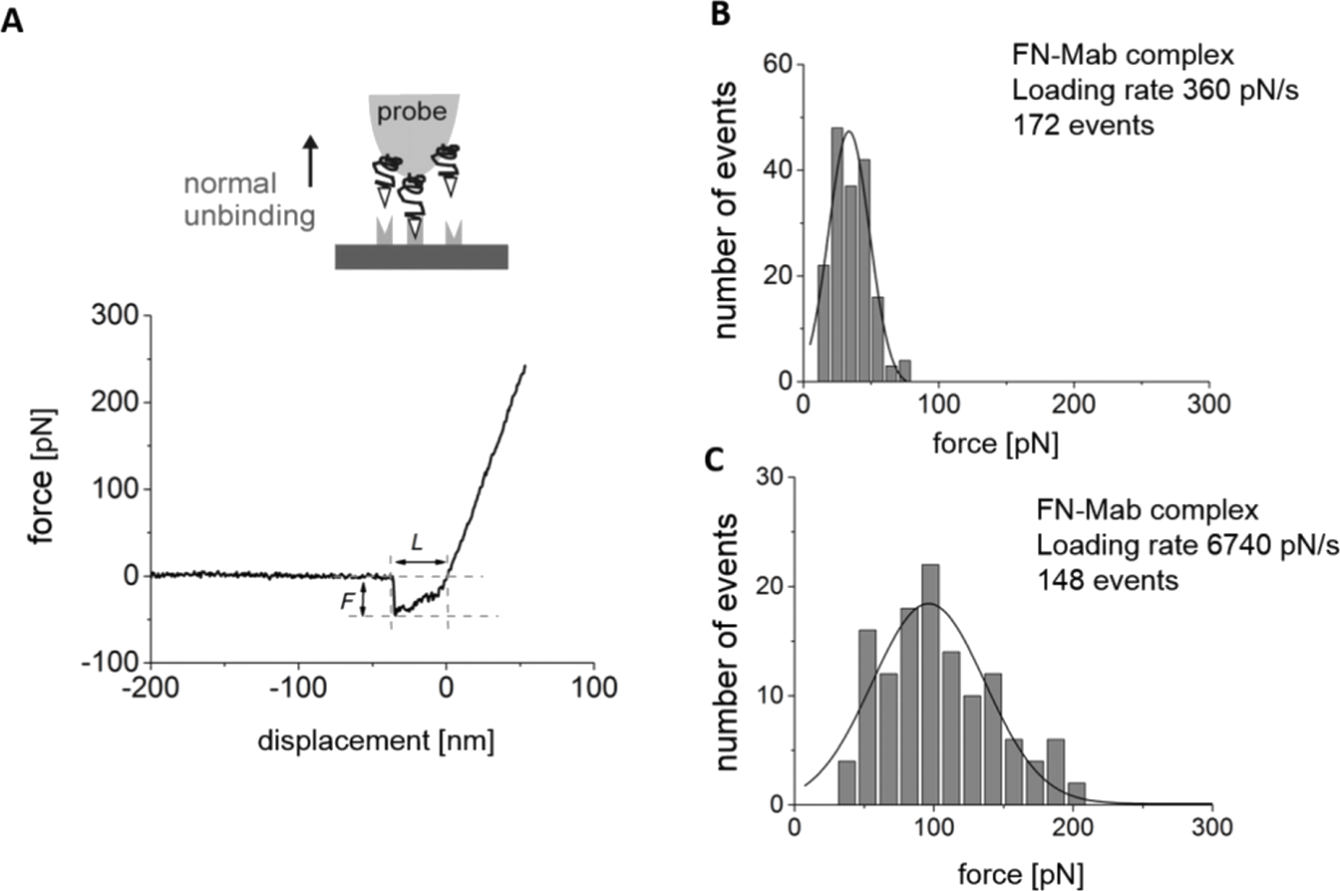
Unbinding of a FN-Mab complex studied using AFM-FS. (A) For each force curve recorded at a given loading rate, the unbinding force, *F*, and unbinding length, *L*, were determined. (B,C) Histograms of unbinding events obtained for two loading rates of 360 pN/s and 6737 pN/s.

In the AFM-FS method, force curves (i.e., the dependence of the cantilever normal deflection converted into force and displacement in the perpendicular direction) were recorded (Figure 4).

For each curve, an unbinding force, *F*, and the length, *L*, were determined (Figure 4A). These two values were used to calculate the effective spring constant, which was used to calculate the loading rate for a given retraction speed. The effective spring constant varied from 0.0030 N/m to 0.0124 N/m for 0.1 µm/s and 10 µm/s, respectively (MLCT type C and MLCT type D cantilevers were used). Then, for each value of the loading rate, a histogram was formed. Exemplary histograms for loading rates of 360 pN/s and 6740 pN/s are presented in Figure 4B,C together with the corresponding Gaussian fit. The fit was used for the determination of the most probable force leading to unbinding of the fibronectin–antibody complex. For all loading rate values, the most probable rupture lengths varied from 10 to 25 nm. The unbinding probability (defined as the ratio between the number of force curves showing the unbinding events and the total number of measured curves) was 20%. After blocking FN by adding free antibody molecules to the solution, followed by 30 min of incubation, the unbinding probability dropped to 5%.

**Figure 5:**
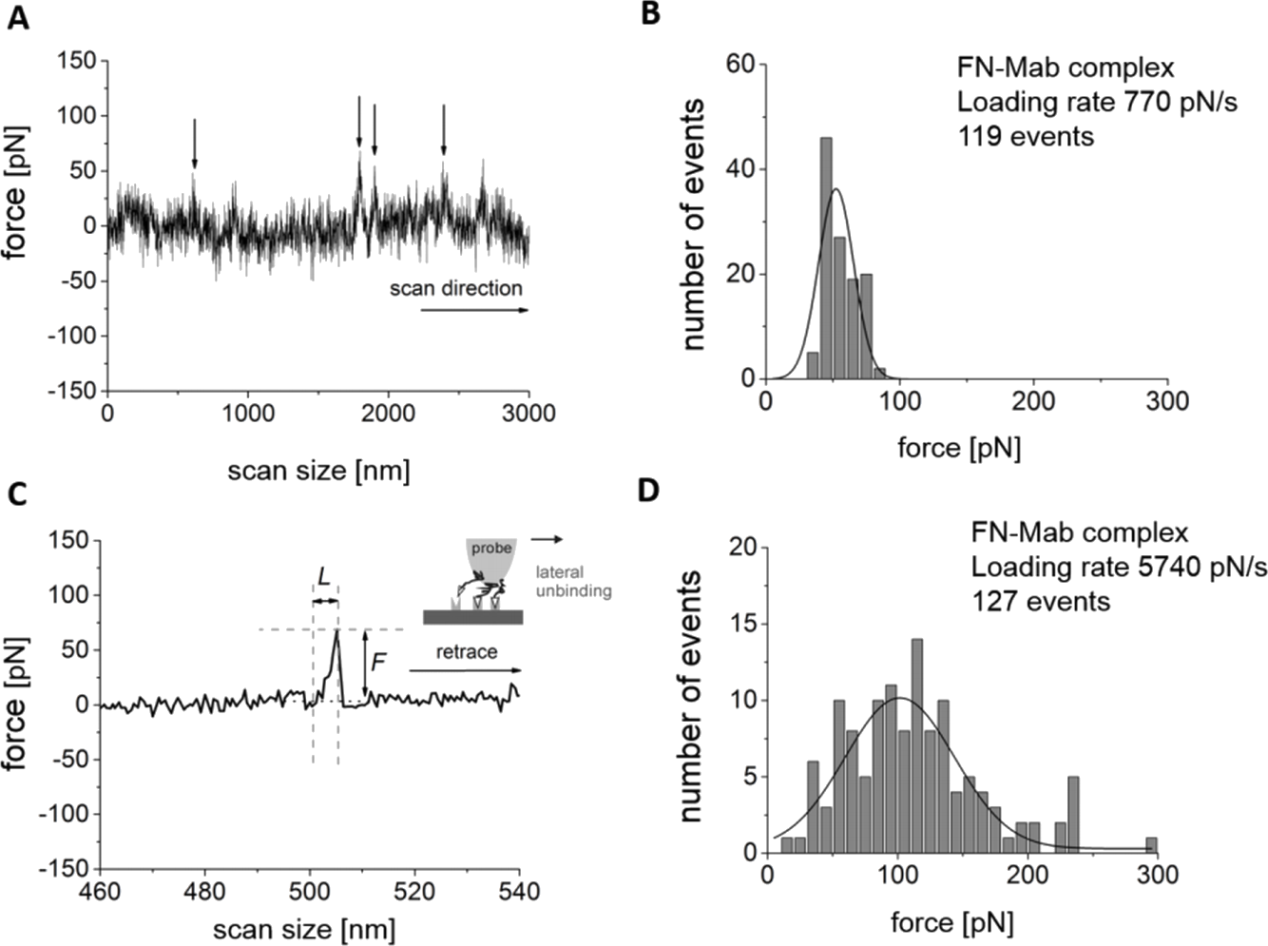
Unbinding of a FN-Mab complex measured by LFM. (A) An exemplary, single LFM signal showing peaks that were attributed to specific interactions between FN-Mab molecules. The arrows indicate events that are suspected to show only the specific interaction. (B) For each spike, an unbinding force, *F*, and an unbinding length, *L*, were determined. (C and D) Histograms of the unbinding force determined for two loading rates, 770 pN/s and 5740 pN/s.

A similar approach was applied within the LFM in order to measure the unbinding forces. Each signal from the torsional cantilever deflection (representing a trace or a retrace) was analyzed in search for sharp peaks. Sharp peaks correspond to unbinding events (ruptures) during a lateral movement of the probing tip functionalized with Mab molecules (Figure 5A). At a higher magnification (each line contains 2048 data points) the unbinding character similar to that observed in the previous AFM-FS experiments (Figure 4A) was revealed.

The unbinding force, *F*, and length, *L*, were determined analogous to the classical AFM-FS measurement (see Figure 5B, where a base line was subtracted for simplicity). Next, *F* and *L* were used to calculate the force required to laterally unbind a single FN-Mab complex. The loading rate in LFM has a similar effect on the most probable unbinding force as in AFM-FS, that is, a higher loading rate value showed a wider distribution of the unbinding force, with its center shifted towards larger force values (Figure 5C). The unbinding probability was calculated as a number of unbinding events divided by the number of points recorded along a single scan line. The resulting unbinding probability was around 6% but less than 10%. Inhibition experiments carried out after 30 min of incubation with a solution containing free antibody molecules showed a remarkable decrease in the unbinding events with a maximum of 1% (only a few peaks were observed in the torsional cantilever deflection signal).

### Loading rate dependence

**Figure 6:**
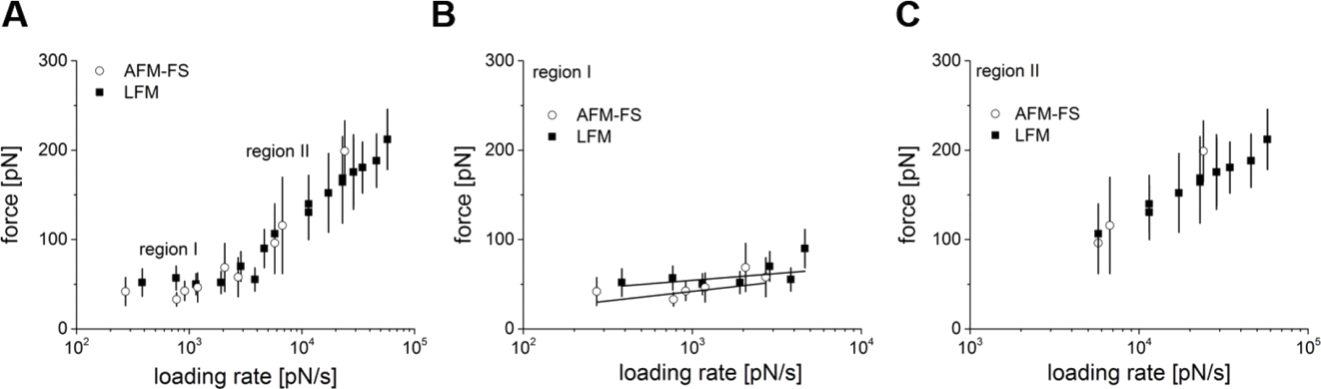
Unbinding force–loading rate dependence obtained for FN-Mab complexes using AFM-FS and LFM. (A) A loading rate dependence obtained for all data showing two regions of linear trends for both applied methods. (B) Region I with a fitted line obtained for lower loading rates. (C) Region II related to higher loading rates values.

The effect of the loading rate on the unbinding force was observed by AFM for many distinct pairs of molecules, bringing deeper insight into the molecular mechanisms of the bond breaking processes [19–22]. For fibronectin interacting with its monoclonal antibody (Clone F-15), our AFM-FS experiments revealed two regimes of loading rates (open dots in Figure 6). A similar trend was observed for the unbinding determined from the torsional cantilever deflection (LFM, black squares in Figure 6).

The experimental points obtained from LFM overlap with those obtained using AFM-FS (Figure 6). Independent of the method used to study the FN-Mab interaction, the presence of two energy barriers can be noticed. Based on the Bell–Evans model, the parameters describing the unbinding process were calculated (Table 1).

**Table 1:** Kinetic parameters derived from the Bell–Evans model and applied to the data obtained by both classical force spectroscopy and lateral force microscopy.

AFM-FS - Classical force spectroscopy			

	*x*_b_ [nm]^a^	*k*_off_ [s^−1^]^b^	τ = 1/*k*_off_ [s]^c^

region I	0.43 ± 0.11	1.18 ± 2.47	0.846
region II	0.06 ± 0.01	20.12 ± 11.52	0.050

LFM - Lateral force spectroscopy			

	*x*_b_ [nm]^a^	*k*_off_ [s^−1^]^b^	τ = 1/*k*_off_ [s]^c^

region I	0.60 ± 0.22	0.53 ± 0.25	1.894
region II	0.09 ± 0.01	11.6 ± 5.51	0.086

^a^*x*_b_ is the position of the energy barrier. ^b^*k*_off_ is the dissociation rate. ^c^τ is the bond lifetime.

Based on the data obtained from AFM-FS, we infer that region I corresponds to the outermost energy barrier located at the position of 0.43 ± 0.11 nm, while region II (see Figure 6) is related to an inner barrier situated at a distance of 0.06 ± 0.01 nm. Qualitatively, similar positions of the energy barriers were observed for the same unbinding process in this complex using the LFM located at 0.60 ± 0.22 nm (the outer barrier, region I) and 0.09 ± 0.01 nm (the inner barrier, region II). The “unphysically” low values of the energy position of the internal barrier (region II) are perhaps related to the limitations of the Bell–Evans phenomenological model. The dissociation rate calculated using both applied methods shows systematically lower dissociation rates in the LFM data for each energy barrier observed (1.18 s^−1^ versus 0.53 s^−1^ and 20.12 s^−1^ versus 11.6 s^−1^, respectively, Table 1).

## Discussion

The unbinding measurements realized by the conventional AFM-FS method are one of the most tedious experiments due to the necessity of high statistics and the low number of unbinding events corresponding to single molecule interactions in a single experimental run [23]. LFM has high potential for performing such experiments in a much more effective way. The first attempt showing that specific interaction forces can be observed in the LFM signal has been applied to lectin–carbohydrate systems [24]. In that work, the specific interactions based on considerations of the frictional forces between a glycoprotein-functionalized AFM probe and a surface modified with lectins were investigated. Such measurements were possible under the assumption that specific forces strongly dominate over non-specific (friction) forces. Moreover, the lack of a reliable and accurate calibration method precluded a comparison of the obtained results with other works carried out for lectin interactions. In our work, we present the direct comparison between the FN-Mab unbinding process measured using LFM and AFM-based force spectroscopies under the assumption that unbinding of molecular complexes is independent of the direction (normal vs lateral [25]). Such a comparison enables the validation of the proposed LFM calibration method and the verification of whether it is possible to obtain similar unbinding characteristics when AFM-FS and LFM methods are applied to study the same type of molecular complex.

To obtain reliable results from the LFM method, the torsion force calibration issue was addressed and a new method was proposed in this paper. Typically, there are two approaches that would deliver either (i) a lateral photodetector sensitivity and a torsional spring constant (in two steps) or (ii) a factor that correlates the raw, uncalibrated signal of the torsional cantilever deflection with the calibrated force value (one step procedure).

The two-step calibration procedure requires separate calibrations of the lateral detector sensitivity and the lateral (or torsional) spring constant. In one approach, a mirrored substrate was tilted and the output voltage was measured as a function of the tilt angle [26]. Alternatively, the lateral sensitivity can be calculated from geometrical considerations or from the initial slope of a friction loop [27]. The angle of the cantilever twist can be estimated assuming that the tip is pinned to the substrate and that the lateral movement has been accurately calibrated. The calibration (determination) of the torsional spring constant is also not an easy task. This can be estimated from an analytical equation in which the cantilever thickness is an essential parameter, usually leading to large errors. Álvarez-Asencio et al. [28] recently proposed a hybrid model to determine the torsional spring constant under the assumption that the normal spring constant can be calibrated using the Sader method [17].

The one-step calibration seems to be much easier to perform since it is based on the direct determination of the friction force without the troublesome separate calibrations of both the lateral photodiode sensitivity and the torsional spring constant [29]. The example of a one-step calibration method has been already presented by Ruan and Bhushan [30]. Here, the cantilever was moved in the direction parallel to its long axis. The friction force was estimated as a product of the vertical spring constant multiplied by the vertical piezoelectric scanner displacement necessary to hold the cantilever deflection constant. The friction measured in this way was later used to calibrate the lateral friction measurements. Unfortunately, this method of calibration, when applied to the FN-Mab system investigated here, delivered unrealistic forces of the order of nN. The methodology of translating a lateral/torsional signal calibration into force units proposed in the current work is similar to that recently developed by Dendzik et al. [15]. However, our method is even more simple and does not require the use of special equipment.

The molecular interactions targeted in this study are between a human FN and monoclonal antibody against FN. An atomic detail basis of a typical model protein–IgG antibody interaction is shown in previous work [25]. The FN is present in the extracellular matrix (ECM) that surrounds living cells in organisms [31]. FN consists of two almost identical monomers linked together by disulphide bridges located close to the carboxyl termini of the monomer [22,32]. The knowledge of the surface topography of the FN molecules deposited on a mica surface enables verification of the quality of protein deposition by direct estimation of single molecule dimensions. Depending on the experimental conditions, fibronectin can be visible either in an elongated or a compact form [22]. Its elongated structure can result in a diameter of about 2.3 nm and a contour length in the range of 120–160 nm, determined for a dimeric FN molecule [33]. Under our conditions, FN was present in a compact, globular form with a mean height of 2.4 ± 0.9 nm. This indicates that after deposition on a mica surface, a layer composed of single molecules was formed. The compact form of FN may be rationalized by considering the electrostatic interaction occurring between different parts of the molecule. Also, the alteration of a protein conformation can be induced by a mechanical deformation during scanning in a contact mode. Interestingly, the average height of 2.4 nm determined by LFM corresponds well with the theoretical value of 2.3 nm estimated by Erickson et al. [34].

Both experimental methods, AFM-FS and LFM, applied here to study the FN–antibody interactions, detected specific unbinding events that were further characterized by two parameters, the unbinding force and rupture length. The inhibition experiments show a significant reduction in the unbinding probability (from 20% to 6%), which indicates the specificity of the interactions. Apparently, when two molecules are pulled apart in the normal direction, they presumably unbind along a different reaction coordinate from the pull in the lateral (inclined) direction. In such a case, one may expect that the energy landscape (and hence the dissociation rate and width of the energy barrier) should be very different. However, it has been theoretically shown that the unbinding of a protein–antibody complex can have a similar character for both modes of enforced “dissociation” [25], illustrating that at initial stages the unbinding proceeds perhaps along the same global reaction pathway, independent of the applied relative pulling force direction.

Another question regarding on the competition between FN unfolding and the unbinding of FN from the antibody arises. Since the whole human FN was used, one can expect that forced unbinding will be also associated with the unfolding (i.e., unfolding may occur when the interaction with the antibody is stronger than the unfolding of the FN domains). Since a sawtooth pattern was not observed in either of the experiments (AFM-FM and FFM), the FN-Mab unbinding in our experiment was weaker than the unfolding of FN domains. The rupture length, at which the unbinding occurred, varied from 10 to 25 nm. This rather high value is probably due to the use of whole, long FN molecules (and not fragments) in our experiments, which could lead to a low-force stretching phase before the unbinding. On the other hand, the calculated rupture length corresponds well to that reported for a similar type of interaction between an antigen and an antibody (i.e., bovine serum albumin and its monoclonal antibody) [35]. Our experiments do not allow for determination of which region (module) of FN interacts with Mab.

As the bond dissociation is a nonequilibrium, dynamic process, accordingly, the rupture force of an isolated bond is not a constant value. Instead, the bond strength is expected to display both a time- and loading-rate-dependent behavior. This has been shown in several experiments where the applied force–loading rate extends over a few orders of magnitude [19–23,35–38]. Figure 5 summarizes the dynamic response of the FN-Mab complex to loading rates between 200 to 70,000 pN/s. Within this range, the unbinding force showed an initial increase of 9.2 ± 3.2 pN and 6.7 ± 5.2 pN for AFM-FS and LFM, respectively. Such a gradual increase is followed by a steep rise starting from a loading rate of about 4000 pN/s. Independent of the loading rate region, the dynamic response curves of the FN-Mab complex overlapped, which may indicate that the activation enthalpy is independent from how the unbinding force is applied. A more detailed analysis of the unbinding of the FN-Mab complex was performed in terms of the Bell model. This analysis shows that both the dissociation rate constant (in the absence of the applied force) and the parameter that characterizes the relative position of the energy barrier are dependent on the mode of rupture (i.e., classical force spectroscopy or lateral force spectroscopy). This likely indicates some differences in the vertical and lateral unbinding scenarios. The computer modelling of a similar unbinding event in an MCP1-IgG antibody complex showed that lateral unbinding forces are about 30% lower than those characteristic of a normal rupture [38]. Regardless of this fact, the loading rate dependence shows two regions within the range of the experimental loading rates. Such observed changes in the slope are usually attributed to the suppression of an outer energy barrier of the energy landscape [19,39]. This suggests that during the unbinding, the single FN-Mab complex goes through a transition state, separating the inner and outer energy barriers. In nearly all molecular complexes studied to date, the dependence of the unbinding force on the logarithm of the loading rate was described by a linear line, indicating the presence of only one energy barrier in the interaction energy landscape. However, in the case of complex molecules, such as proteins, the kinetic processes can be characterized by multiple local maxima and minima in the interaction potential along the reaction coordinate. In these situations, the plot of the most probable unbinding force versus the logarithm of the loading rate displays a sequence of lines with different slopes, each corresponding to the position of a particular energy barrier.

## Conclusion

The force measurements carried out for a fibronectin–antibody complex showed similarity in the unbinding process, independent of how the rupture force was applied by the AFM cantilever movement: either normal (AFM-FS) or lateral (LFM). The relation between the measured unbinding force and the loading rate applied overlapped for the AFM-FS and LFM methods. These findings demonstrate that the detection of specific protein–protein forces using lateral force microscopy (LFM) is possible. However, the appropriate calibration suitable for LFM must be performed and the assumption that specific forces dominate over non-specific must be fulfilled. In this work we presented an effective variant of the calibration of the cantilevers for the LFM measurements. Our findings on the FN-Mab antibody protein complex validate the proposed novel and simple method of a lateral signal calibration. Thus, it can be foreseen that the lateral scanning of the sample could accelerate an unbinding measurement as compared to the conventional AFM molecular recognition study. We anticipate that the LFM technique will be useful since it is not limited to proteins or biological samples; however, more experiments are needed to better understand the limitations/advantages of the use of LFM in molecular recognition processes.

## Experimental

### Proteins

Fibronectin from human plasma (*M*_w_ ≈ 450 kDa, Sigma) was used in all experiments. The fibronectin was detected by the use of monoclonal antibody against human fibronectin (Mab, Clone FN-15, Sigma), produced in mouse ascites fluid after immunization of the mice with fibronectin isolated from human plasma.

### Other reagents

Other reagents used in the experiments were: (a) phosphate buffered saline (PBS, ICN Biomedicals, pH 7.4, containing 10 mM of PO_4_^2−^, 137 mM of NaCl and 27 mM of KCl) was used to prepare all protein solutions; (b) 3-aminopropyltriethoxysilane (APTES, Sigma) was used for the silanization of the mica and cantilever surfaces; (c) 2.5% glutaraldehyde aqueous solution, prepared from a 25% solution of glutaraldehyde was purchased from Sigma. All solutions were prepared using deionized water (Cobrabid water purification system, 0.08 µS).

### Cantilevers

Commercially available cantilevers (MLCT-AUHW, gold coated, not sharpened) purchased from Veeco were used. For all experiments, the cantilever type C was chosen. It is characterized by the nominal spring constant of 0.01 N/m, a resonant frequency of 7.0 kHz, and geometrical dimensions of 320 µm (length), 22 µm (width) and 0.6 µm (thickness). The open angle of a tip pyramid was 35° while the radius of curvature was 50 nm. As a reference, the cantilever type B was used. It is a rectangular-shaped cantilever with geometrical dimensions of 210 µm (length), 20 µm (width) and 0.6 µm (thickness). It is characterized by a nominal cantilever spring constant of 0.02 N/m and a resonant frequency of 10 kHz.

### Fibronectin deposition on mica surface

As a support for the deposition of fibronectin, a modified mica surface was used. First, freshly cleaved mica was silanized with APTES. The APTES was deposited on the mica surface from gas phase for 2 h in a desiccator. Next, the sample was immersed in 2.5% glutaraldehyde aqueous solution for 20 min and afterwards rinsed with 10 mM PBS buffer. Then, the prepared sample was completely immersed in 0.1 mg/mL FN solution in PBS for 60 min, which prevented drying. Then, it was gently rinsed with PBS and immediately measured by AFM-FS or LFM.

### Cantilever functionalization with Mab

The cantilevers (MLCT, type C, Veeco) used for both the AFM-FS and LFM measurements were modified using the same protocol as for the mica surface. Similar to mica surface, the cantilevers were silanized using APTES from the gas phase, then their surface was activated using a 1.5% aqueous glutaraldehyde solution and rinsed with PBS buffer. Then, the cantilevers were immersed in a drop (≈50 µL) of PBS solution of 0.05 mg/mL Mab for 30 min, and afterwards rinsed with PBS buffer. These prepared cantilevers were immediately used in the measurements.

### Atomic force microscope

All measurements were carried out using commercially available devices (PSIA XE100 and XE120, Park Systems, Korea) equipped with a “liquid cell” setup, in 10 mM PBS buffer. The surface topography of a fibronectin-coated mica surface was measured in contact mode over an area of 10 × 10 µm, with set point of 0.2 nN and scan rate of 0.8 Hz.

### Unbinding experiments

In AFM-based classical force spectroscopy, the unbinding forces of the interaction between fibronectin (FN) and monoclonal antibody against FN (FN-Mab) were measured using a fibronectin-coated surface and antibody-modified cantilevers always prepared in the same way. The measurements were carried out seven times, each time with a fresh AFM probe and a new sample (newly Mab-coated cantilever and freshly deposited fibronectin on the mica surface). These experiments were carried out using two cantilever types, MLCT-C and MLCT-D, characterized by nominal spring constants of 0.01 N/m and 0.03 N/m, respectively.

In lateral force microscopy, the cantilever (MLCT-C; nominal spring constant of 0.01 N/m) was moved laterally over the mica surface covered with fibronectin. The friction images always contained 2048 points per line. Three scan sizes from 1 to 6 µm were recorded at a scan rate from 0.1 Hz to 10 Hz (the scan velocity varied from 0.1 to 60 µm/s). In the classical AFM-FS experiment, the cantilever, localized in one selected point over the sample surface, was moved perpendicularly towards the FN-functionalized mica surface, followed by retraction. During this movement, the normal cantilever deflection was recorded as a function of relative scan position (i.e., force curves were collected). The force curves (2048 points per cycle, for approach and retract) were recorded as a function of retraction speed. The velocity was in a typical range of AFM retraction velocity and varied from 0.1 to 10 µm/s.

### Specificity of the interaction

To assure the specificity of the interaction, the fibronectin was blocked using monoclonal antibody, same as that used for the cantilever functionalization. The inhibition experiments were carried out after 30 min of incubation with the PBS solution containing free antibody molecules. Afterwards, samples were rinsed with PBS buffer and immediately measured. The same protocol for the interaction inhibition was used in both types of experiments (AFM-FS and LFM).

### Bell–Evans model

During the AFM unbinding, the external forces applied to a protein–ligand complex pull the ligand off of its initial position in the binding pocket. If the transition from bound to unbound states over the energy barrier is associated with a displacement in the direction of the acting force, the height of the energy barrier is lowered by the term *F∙x*_b_ where *x*_b_ is the difference between the bound and unbound states [38–40]. In 1997, Evans and Ritchie introduced a model describing the bond rupture under an external force [41] for the case when the applied force *F* changes linearly in time *t* according to:

[1]
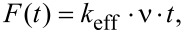


where *k*_eff_ is the effective spring constant accounting for the AFM cantilever and the single bond spring constants, and ν is the tip velocity. The dependence of the unbinding force on the loading rate is given as [41]:

[2]
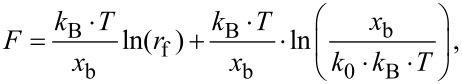


where *k*_B_ is Boltzmann’s constant, *T* is the temperature, *x*_b_ is the difference between the maximum of the energy barrier from the potential minimum, *k*_0_ is the dissociation rate of the unbinding process, and *r*_f_ is the loading rate defining how fast an external force changes as a function of time.
